# Adaptive efficient compression of genomes

**DOI:** 10.1186/1748-7188-7-30

**Published:** 2012-11-12

**Authors:** Sebastian Wandelt, Ulf Leser

**Affiliations:** 1 Institute for Computer Science, Humboldt-Universität zu Berlin, Berlin, Germany

**Keywords:** Sequence compression, Referential compression, Heuristics, Scalability

## Abstract

Modern high-throughput sequencing technologies are able to generate DNA sequences at an ever increasing rate. In parallel to the decreasing experimental time and cost necessary to produce DNA sequences, computational requirements for analysis and storage of the sequences are steeply increasing. Compression is a key technology to deal with this challenge. Recently, referential compression schemes, storing only the differences between a to-be-compressed input and a known reference sequence, gained a lot of interest in this field. However, memory requirements of the current algorithms are high and run times often are slow. In this paper, we propose an adaptive, parallel and highly efficient referential sequence compression method which allows fine-tuning of the trade-off between required memory and compression speed. When using 12 MB of memory, our method is for human genomes on-par with the best previous algorithms in terms of compression ratio (400:1) and compression speed. In contrast, it compresses a complete human genome in just 11 seconds when provided with 9 GB of main memory, which is almost three times faster than the best competitor while using less main memory.

## Background

The development of novel high-throughput DNA sequencing techniques has led to an ever increasing flood of data. While it took roughly 12 years and an estimated amount of 3 billion USD to decipher the first human genome [1], current techniques, usually summarized under the term second generation sequencing (SGS), are able to produce roughly the same amount of data in about a week at a current cost of roughly 2000 USD. On top, third generation sequencing promises to deliver a further speed-up, reducing the time and price for sequencing a human genome from weeks to days and from thousands to under a hundred USD, respectively [2].

Large-scale projects are generating comprehensive surveys of the genomic landscape of various diseases by sequencing thousands of genomes [3]. Managing, storing and analyzing this quickly growing amount of data is challenging [4]. It requires large disk arrays for storage, and large compute clusters for analysis. A recent suggestion is to use cloud infrastructures for this purpose [5-7]. However, before being analyzed in a cloud, data first has to be shipped to the cloud, making bandwidth in file transfer one of the major bottlenecks in cloud-based DNA analysis [8]. Accordingly, sequence compression is a key technology to cope with the increasing flood of DNA sequences [9-11].

To store a complete genome of a human being, one needs roughly 3GB (uncompressed). Substitutional or statistic compression schemes can reduce the space requirements by up to 6:1 (one base is encoded with up to 1.3 Bit) [12,13]. However, in many projects only genomes from one species are considered. This means that projects often deal with hundreds of highly similar genomes; for instance, two randomly selected human genomes are identical to an estimated 99.9%. This observation is exploited by so-called referential compression schemes, which only encode the differences of an input sequence with respect to a pre-selected reference sequence. Using space-efficient encoding of differences and clever algorithms for finding long stretches of DNA without differences, the best current referential compression algorithm we are aware of reports a compression rates of up to 500:1 for human genomes [14].

However, all existing compression schemes have in common that they have very high demands on the underlying hardware (up to 25 GB, for instance [15,16]). Furthermore, the time needed for compressing the amount of sequences corresponding to a human genome may be up to several hours, which easily out-weights the time savings during file transfer. Furthermore, existing approaches cannot easily be parallelized to reduce compression time, because such a parallelization requires to share information about the generated compression model. This communication/synchronization overhead may easily mitigate the positive effects gained by parallelization.

In this paper, we present an adaptive, scalable and highly efficient algorithm for referential compression of DNA sequences genomes. It is able to gracefully trade-off compression time and space requirements while consistently achieving very good compression rates. For instance, our algorithms requires only 12 MB memory to compress/decompress a human genome at the same speed as the fastest existing approach. Using 9 GB of memory usage, our method performs up to three times faster than the best competitor while still needing less main memory. Both variants achieve similar compression rates of approximately 400:1 for human DNA. For yeast sequences, the compression ratios are lower, since two yeast genomes can have substantial differences.

The remaining part of this paper is structured as follows. We first explain the main ideas behind our algorithm in Section “General Idea”. The concrete design choices and algorithms are described in detail in Section “Genome Compression”. We discuss related work in Section “Related Work”, before we provide an evaluation of our method in Section “Evaluation”. The paper is concluded with Section “Conclusions”.

## General Idea

We denote strings with *s,t*. The length of a string *s* is denoted with |*s*| and the substring starting at position *i* with length *n* is denoted *s*(*i,n*). *s*(*i*) is an abbreviation for *s*(*i*,1). All positions in a string are zero-based, i.e. the first character is accessed by *s*(0). The concatenation of two strings *s* and *t* is denoted with *s*∘*t*. Although a genome can be encoded with four characters, i.e. A,C,G, and T, we allow arbitrary symbols. For instance, symbol *N* is often used to indicate an unknown base. Given two strings *s* and *t*, the *longest prefix-suffix match of s* in *t*, is the longest string *t*_*m*_, such that *t *=* t*_1_∘*t*_*m*_∘*t*_2_ and *s*(0,|*t*_*m*_|) =* t*_*m*_.

We want to compress a given input genome with respect to a reference genome, by only encoding differences between the input and the reference. This yields loss-less compression, i.e. based on the reference genome and the difference description it is possible to recover the input genome. The idea is to split up a given reference genome into blocks. In general, two genomes of the same species are very similar to each other, and blocks are chosen in a way that long matches of to-be-compressed blocks can often be found in reference blocks by local search. Please note that splitting the reference into blocks of fixed length is not an optimal way, since different sizes of the blocks can provide better performance in terms of compression ratio. However, for the sake of compression speed and easier data handling, we only consider a fixed block length.

For each reference genome block, a suffix tree is computed. The suffix trees allow to find longest prefix-suffix matches of parts of the input genome and the reference genome efficiently. The compression process is informally summarized in Algorithm 1.

### Algorithm 1: Sketch of our Compression Algorithm

1: **while** Input contains characters

2: Find matching reference block *B* for current input position

3: Perform referential compression with respect to *B* until we cannot find “long” matches anymore

4: **end while**

The compression algorithm generates a set of referential matches with respect to reference blocks. The output of our compression algorithm is a compressed file of entries, such that each entry is one of the following: 

• **Block-change entry ***BC*(*i*): next entries are encoded with respect to reference block *i*.

• **Relative match entry ***RM*(*i,j*): The input matches the reference block at position *i* for *j* characters.

• **Raw entry ***R*(*s*): A string *s* is encoded raw (for instance if there is no good matching block).

**Figure 1:**
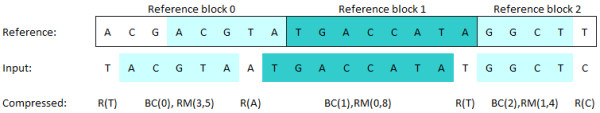
**Example for relative compression.** An example compression is given in Figure 1. An input sequence “TACGTAAT..” is compressed with respect to a reference sequence “ACGACGTA..”. The first character of the input is encoded raw, with entry *R*(*T*), then the first reference block is chosen, with entry *BC*(0) , in order to referentially encode the subsequence “ACGTA”, with entry *RM*(3,5).

An example compression is given in Figure 1. An input sequence “TACGTAAT..” is compressed with respect to a reference sequence “ACGACGTA..”. The first character of the input is encoded raw, with entry *R*(*T*), then the first reference block is chosen, with entry *BC*(0) , in order to referentially encode the subsequence “ACGTA”, with entry *RM*(3,5).

Please note that block change entries can be actually avoided, since local positions in a block can be easily transformed into global positions in the reference. In the remaining part, we will still assume the usage of block change entries.

## Genome Compression

### Index Construction

In order to find matches of the input in reference blocks, we need an index structure over the reference blocks. Suffix trees [17] are tree data structures which allow for fast access to all suffixes of a given string. Each suffix is usually represented by a path from the root of the tree to a leaf. Please note that the longest prefix-suffix match problem of *s* in *t* can be be easily solved by depth-first search given a suffix tree for the string *t*. The focus recently shifted towards so-called *compressed suffix trees*. One example of a compressed suffix tree implementation is CST++ [18], which uses a new approach to compress the longest common prefix-array and achieves, depending on the sampling rates of the (inverse) suffix array, an usual space overhead of (4−6)∗*n* bits. In the following, the compressed suffix tree of a string *s* is denoted with *CST*(*s*).

In addition, we need a reference genome for our compression algorithm. The reference sequence is given as a set of compressed FASTA-files, one for each chromosome. The index generation algorithm iterates over all chromosomes of the reference genome. Each chromosome is split up into blocks of a maximum size *BS*. For instance, given the textual representation *s*_1_ of Chromosome 1, we compute *m* substrings *b*_1,1_,…,*b*_1,*m*_, such that 

• *s*_1_ =* b*_1,1_,…,*b*_1,*m*_,

• ∀*i *≤ (*m*−1):|*b*_1,*i*_| =* BS*, and

• [*b*_1,*m*_] ≤* BS*.

We do the same for all other chromosomes of the reference genome. For each reference block a compressed suffix tree is computed and stored on hard disk together with the raw reference block. In addition, we keep track of the order of the blocks, which is induced by their position on the chromosomes. This meta information is used below for optimizing our compression algorithm.

The memory consumption during index creation is limited as follows: at each step of the index generation we have one raw reference block of size at most BS bytes in main memory plus (roughly) 4∗BS bytes for its compressed suffix tree. For example, given a BS of 4 MB, the main memory usage can be restricted to approximately 20 MB. The maximum value for BS is 300 MB, since the largest human chromosome (Chromosome 1) has about 247 million nucleotide base pairs, and each base is encoded as one byte.

Please note that we could have encoded bases of the reference genome with 3 bits (five entries: A,C,G,N,T), in order to further reduce memory usage. However, our tests indicate that the space saving yields a significant time overhead for accessing bases. Furthermore, the 3-Bit-encoding would not allow us to compress sequences against references with other symbols than these five.

### Compression and Decompression

In the following, we present our compression algorithm for genome sequences in detail. Algorithms do not show range checks for the sake of readability. Algorithm 2 assumes the input genome in *Input* (as a string of bases). The input string is traversed from left to right, and depending on the current characters in the input and in the reference block, different subroutines are executed.

### Algorithm 2: Compression Algorithm

1: *P*_*in*_←0

2: *P*_*raw*_←0

3: FIND-MATCH

4: **while ***P*_*in *_≠ ∣*Input*∣**do**

5: **if ***Input*(*P*_*in*_) =* B*(*P*_*raw*_)** then**

6: ENCODE-REF

7: **else if ***Input*(*P*_*in*_)∉{*A*,*C*,*G*,*T*}** then**

8: ENCODE-RAW

9: **else**

10: FIND-MATCH

11: **end if**

12: **end while**

In the beginning of the compression algorithm, a match for the current input position in the reference is searched, using function FIND-MATCH, Algorithm 3, explained in detail below. If the current reference block *B* matches the input at the current position, then we try to generate a (as long as possible) reference match in function ENCODE-REF, Algorithm 4. If the current input base does not equal the current reference block base, and the input is not a *normal* base, i.e. neither *A*, *C*, *G*, or *T*, then the base and all the following non-normal bases are added as raw entries to the compressed genome file in function ENCODE-RAW, Algorithm 5. Finally, if neither of the conditions is satisfied, then the algorithm tries to find a new match (either in the current block or in another reference block) using function FIND-MATCH.

### Algorithm 3: FIND-MATCH Function

1: *Max*←25

2: **if** not HAS-LOCAL-ALTERNATIVE() **then**

3: **for all***B*_*j*_∈*Blocks***do**

4: **if***m*(*Input*,*P*_*in*_,*B*_*j*_)≥*Max***then**

5: *Max*←*m*(*Input*,*P*_*in*_,*B*_*j*_)

6: *B*←*B*_*j*_

7: **end if**

8: **end for**

9: **if***Max*≤25**then**

10: *Raws*←*Input*(*P*_*in*_,*Max*)

11: Add R(*Raws*) to output

12: *P*_*in*_←*P*_*in*_ + *Max*

13: **else**

14: *P*_*raw*_← beginning of the longest match in *B*

15: Add BC(*B*) to output

16: **end if**

17: **end if**

### Algorithm 4: ENCODE-REF Function

1: *M*←0

2: **while***Input*(*P*_*in*_)=*B*(*P*_*raw*_)**do**

3: *M*←*M* + 1

4: *P*_*in*_←*P*_*in*_ + 1

5: *P*_*raw*_←*P*_*raw*_ + 1

6: **end while**

7: Add RM(*P*_*raw*_-M,*M*) to output

### Algorithm 5: ENCODE-RAW Function

1: *Raws*←*""*

2: **while***Input*(*P*_*in*_)∉{*A*,*C*,*G*,*T*}**do**

3: *Raws*←*Raws*∘*Input*[*P*_*in*_];

4: *P*_*in*_←*P*_*in*_ + 1;

5: **end while**

6: Add R(*Raws*) to output

In Algorithm 4 the number of matching characters between current input position and current reference position are determined and stored in variable *M*. A reference entry RM(*P*_*raw*_-M,*M*) is added to the compressed output. The encoding of a raw sequence in Algorithm 5 is straight-forward: The string *Raws* is filled with bases from the input until a *normal* base is found and R(*Raws*) is added to the output.

Algorithm 3 requires more thoughts. The function FIND-MATCH is called, whenever there is a mismatch between the input and the reference. In this case, we need to find an alternative match for the current input position. First, we check whether there exists a match in the neighbourhood (mutations caused by few single nucleotide polymorphisms) of the current position of the reference block. The process is explained in detail later. If we cannot find a local match, then all the reference blocks are checked for a better match. The expression *m*(*Input*,*P*_*in*_,*B*_*j*_) returns the position of the longest prefix-suffix match of the current input in *B*_*j*_.

In our implementation of Algorithm 3, we care about the order in which all reference blocks are traversed. This is necessary, since loading blocks from the disk is a time consuming effort and should be avoided. Reference blocks are traversed with the following heuristic: 

1. The block left and right of the current reference block

2. All other blocks on the same chromosome as the current reference block

3. All other blocks of the reference genome

If there is no long enough match in any reference block, then we just encode few raw bases. Matches in other blocks are required to be longer than 25 characters, in order to avoid random matches. Our experiments have shown that the mere length of the human DNA causes a lot of unrelated matches with less than 20-25 characters.

Algorithm 3 uses the function *HasLocalAlternative*. The intuition is that we want to avoid searching all possible reference blocks all the time. Our experiments showed that one longest prefix-suffix-match lookup-operation in a single compressed suffix tree can take up to few milliseconds depending on the size. This is basically caused by the high bit-wise compression of the reference genome. Furthermore, potentially we have to load all the other reference block’s CTSs from the hard disk. Therefore, the naive strategy to search for a new reference block on *each mismatch* does not scale.

Instead we use a local search in the neighbourhood of the current input position and of the current reference position. This strategy has a biological foundation: Often two parts of a genome might only be different by few bases (base insertion, base removal, of base mutation). Whenever we find an appropriate match in the neighbourhood, we avoid checking other reference blocks, although they might contain better matches. In fact, if we searched for the best reference blocks each time, we could increase the compression rate slightly for the price of being orders of magnitude slower.

The algorithm for local neighbourhood search is shown in Algorithm 6. If a match of at least 20 characters can be found near the current input and reference positions, then the difference until the match is encoded raw, and the match is encoded referentially.

Decompression of the relative genome data is straightforward. Basically, all entries are processed according to their type. For decompression of genome data we do not need the compressed suffix trees any more, but only the raw blocks of the reference genome. The decompression of genome data is the least CPU-intensive task. Our evaluation in the next section shows that index generation and compression are CPU-bound, while the decompression phase is I/O-bound.

### Algorithm 6: HAS-LOCAL-ALTERNATIVE Function

1: **for***i*∈{0,1,…,6,7}**do**

2: **for***j*∈{−7,−6,…,6,7}**do**

3: **if***Input*(*P*_*in*_ + *i*,20)=*B*(*P*_*raw*_ + *j*,20)**then**

4: **if***i*≥1**then**

5: *Raws*←*Input*(*P*_*in*_,*i*)

6: Add R(*Raws*) to output

7: *P*_*in*_←*P*_*in*_ + *i*

8: **end if**

9: *P*_*raw*_←*P*_*raw*_ + *j*

10: ENCODE-REF

11: RETURN

12: **end if**

13: **end for**

14: **end for**

We emphasize that our compression scheme is a heuristic, which mainly works when compressing a sequence with respect to a reference from the same species. The efficiency of this heuristic is evaluated in Section “Evaluation”. The worst-case time complexity of our compression algorithm is *O*(—*Input*—), since we need to traverse the whole input sequence one time from left to right and for each character we perform (in the worst case) one lookup in the reference. The complexity of the lookup depends on the length of the substring being looked up (we assume a fixed maximal match length) and is therefore constant. The worst-case space complexity is *O*(—*Input*—). Each character of the input is stored at most one time: either inside a raw block or as part of a referential block.

## Related Work

In the following, we review existing work on biological data compression. In general, compression algorithms are either substitutional, statistical, or referential. Substitutional algorithms [19] replace long repeated substrings by references, e.g. Lempel-Ziv-based compression. Statistical algorithms [13,20] derive a predictive model from (a subset of) the input, based on partial matches. If the model always indicates high probabilities for the next symbol, then high compression rates are possible. While referential algorithms replace long substrings as well, the source for these subsets is usually not part of the input (the sequence to be compressed). Referential compression algorithms have drawn a lot of attention recently, since they allow for very high compression rates.

In [16], RLZ, a self-indexing based approach is proposed as follows: Given a self-index for a base sequence, compress other sequences with LZ77 encoding relative to the base sequence. In fact, a suffix array for the reference sequence is built and the reference entries are position references with length into the base sequence. Please note that the authors do not store raw sequences at any time, but only encode based on a list of references, no matter how long.

The authors of [21] proposed to store human genomes relatively to a reference chromosome. They used variable integers for storing absolute and relative positions of matches and represent often used *k*-mers (sequences of length *k*) with Huffman encoding.

In [22], a LZ77-style compression scheme is proposed. The main difference is that several reference sequences are taken into account. The match-finding process is based on hashing. Compression is performed on input blocks with shared Huffman models, in order to support random access. In [23], another LZ77-style compression scheme is proposed.

There exists further early work on non-referential compression algorithms [24-27]. Furthermore, there is compression based on context-free grammars [28], determination of Markov-model based probabilities of sequence positions [29], and hybrid methods [30]. In [12], a splay tree-based compression algorithm is proposed, which favors encoding of recently seen symbols. Finally, there exists previous work on relative compression of reads and alignment data, e.g. [31,32].

## Evaluation

In the following section, we evaluate our proposed compression scheme. All experiments have been run on a Acer Aspire 5950G with 16 GB RAM and Intel Core i7-2670QM, on Fedora 16 (64-Bit, Linux kernel 3.1). The code was implemented in C++, using the BOOST library [33], CST [34], and libz. All size measures are in byte, e.g. 1 MB means 1,000,000 bytes. The source code is available for download^1^.

First, we performed compression tests on human genomes. Our set of data genomes consists of 1000 genomes from the 1000 Genome project [35]. The 1000 Genome project group provides all sequenced genomes in Variant Call Format (VCF) [36] for download^2^. The Variant Call Format describes differences of sets of genomes with respect to a reference sequence, based on SNPs and indels. We have extracted one consensus sequence each for in total 1000 genomes. Since the project contains slightly more than 1000 genomes, we have only extracted the first 1000 genomes (columns from left to right) named in these VCF files. The consensus sequences for each chromosome of each genome were stored GZip-compressed on a hard disk. These 1000 GZip-compressed genomes need in total 700 GB of storage.

### Compression Ratio

**Figure 2:**
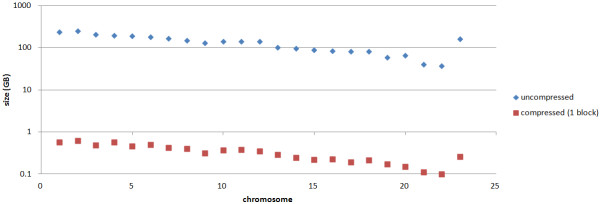
**Compressed file size for different input chromosomes(in MB).** We have compressed each chromosome referentially against the reference chromosome taken from HG19 [37]. The results are shown in Figure 2. The overall compression ratio obtained for the 1000 human genomes is 397:1.

First, we have compressed each chromosome referentially against the reference chromosome taken from HG19 [37]. The results are shown in Figure 2. The first chromosome of 1000 humans needs 236.6 GB of uncompressed storage, while the referentially compressed file needs only 0.57 GB, yielding a compression ratio of 415:1. The smallest human chromosome of our 1000 genomes, Chromosome 22, is compressed from 36.4 GB down to 0.1 GB, yielding a compression ratio of 364:1. The overall compression ratio obtained for the 1000 human genomes is 397:1.

For very similar sequences we achieve higher compression rates, than for less related sequences. This is inherent to all referential compression schemes: the more similar input and reference sequence are, longer referential matches can be found.

**Figure 3:**
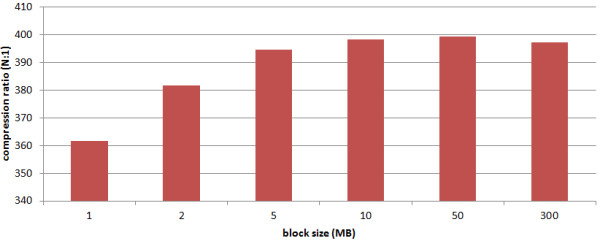
**Compression ratio by block size.** We have evaluated the impact of the block size on the compression ratio. The results (average over all human genomes and chromosomes ) are shown in Figure 3. In general, a larger block size will yield better compression ratios.

We have evaluated the impact of the block size on the compression ratio. The results (average over all human genomes and chromosomes ) are shown in Figure 3. In general, a larger block size will yield better compression ratios. The intuition is that a larger block in main memory allows for finding longer matches. However, for a block size of 1 MB, our compression scheme can still achieve a compression ratio of 361:1, which is roughly competitive to existing relative compression schemes for human genome sequences. RLZ obtains a compression ration of 80:1 and RLZopt a compression ratio of 133:1 for human genomes. GDC achieves compression ratios of 200:1 - 500:1, depending on speed-tradeoffs. There exists one variant GDC-ultra, which achieves a compression ratio of 1000:1, which switches the reference sequence during compression. Switching the reference naturally allows for higher compression ratios. However, since we only use one reference, it seems to be fair to only compare our results to the non-ultra variant of GDC, obtaining a compression ratio of at most 500:1.

Please note that the compression ratio for 300 MB is actually smaller than for 50MB. One explanation could be that all these matches found with smaller blocks allow for a shorter encoding than the matches found in a longer block. At first sight this might sound counter intuitive. To the best of our knowledge, no research has been conducted in this area, since the compression gain mainly depends on the choice of referential entries, i.e. how to encode positions, length entries and raw base entries. Referential compression is an optimization problem, where the longest matches often, but not necessarily, yield the shortest compressed representation. For instance, sometimes more (shorter) matches can be encoded more efficiently than less (longer) matches. We think that these effects are important to be studied in Future Work.

The overall index size for the reference sequence (per chromosome) is in average 202 MB, while an average uncompressed input sequence is roughly 130 MB long. The size of the index structure is decreasing with decreasing block size, since the maximum length of paths in the suffix tree is limited.

Additional experiments have been conducted on yeast genomes. We have downloaded^3^ 39 yeast genomes, have chosen an arbitrary reference sequence (273614N) and referentially compressed the other 38 genomes (all chromosomes concatenated to each other) with respect to the reference. The average compression ratio is 61:1. The lower compression ratio compared to human sequences is not surprising, since it is well known that two yeast genomes can be less similar than even a human genome and a chimpanzee genome. In these cases (unoptimized) referential compression schemes do not obtain such nice compression ratios as with human genomes. RLZopt obtains compression ratios of 50:1, and GDC obtains compression ratios of 70:1-100:1, depending on speed-tradeoffs.

In average, the index size of an average yeast gnome (around 12 MB) was found to be around 17.5 MB.

### Compression Times

**Figure 4:**
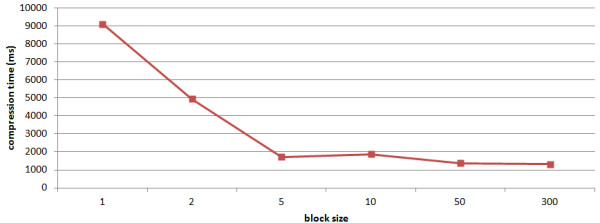
**Compression ratio by block size.** The results for serial compression of the 1000 human genomes are shown in Figure 4. All the results were averaged over all chromosomes as input. Clearly compression time decreases with increasing block index size, since less time is spent on traversing other blocks to find best matches.

For serial compression, the compression algorithm performs compression on one block at a time (in a single thread). The memory requirements during compression are roughly 6*BS (one reference raw block, one input genome block, and the compressed suffix tree of the input block). In addition we use a write buffer for output operations (size: 1 MB), which can be neglected. The results for serial compression of the 1000 human genomes are shown in Figure 4. All the results were averaged over all chromosomes as input.

Clearly compression time decreases with increasing block index size, since less time is spent on traversing other blocks to find best matches. If the block index size is 1 MB (equals to roughly 6 MB main memory usage), the compression times are roughly 9 seconds per chromosome, i.e. around 200 seconds to compress a whole human genome. This yields a compression speed of around 15 MB/s. With a block index size of 300 MB (roughly 1.8 GB main memory usage), the compression time is down to 1.5 seconds per chromosome, i.e. around 35 seconds for compression a whole human genome. This yields a compression speed of 85 MB/s. The time necessary to create the compressed suffix tree for the reference is roughly 20 minutes. If one takes the position that indexing time should belong to the online search time, then the compression speed is reduced to 2.42 MB/s for a single genomes and to 82.87 MB/s for 1000 genomes. However, we are convinced that index construction should be taken as an offline task.

RLZopt obtains a compression speed of 1.34 MB/s, while GDC achieves compression speeds of 4-35 MB/s, depending on speed-tradeoffs. Therefore, our adaptive algorithm seems to be competitive with existing relative compression schemes, while using less memory in a controllable (by changing the block size) way.

In addition, we have performed experiments with 39 yeast genomes as in the previous subsection. The compression time is around 70 seconds for one yeast genome, yielding a compression speed of 0.17 MB/s. As expected, our local search heuristic does not work as good as with human genomes. Most of the time is spent on consulting the compressed suffix tree which often only yields very short matches of length 10-20. RLZopt has a compression speed of 1.6 MB/s and GDC achieves compression speeds of 2-33 MB/s, depending on speed-tradeoffs. In this case, it seems to be advantageous to use a hash table as an index structure instead of a suffix tree, as proposed by the authors of GDC.

The parallelization on multi cores of our approach is straightforward. Block-processing can be easily distributed on several CPUs (or even machines). However, it has to be kept in mind that each compressor might work on different parts of the reference genome, which means that for *n* compressor threads the memory usage is increased by the factor *n*. The upper limit main memory usage for seven threads is 7 GB (maximum size of the complete index) + 7∗*BS*, if all index structures are loaded into main memory.

Further investigation showed that the compression time in the latter case is dominated by loading the index files into main memory. Basically, in the beginning each thread requests a set of index blocks, which (due to parallel access) might be loaded in a partial random access manner.

To counter act this effect, we have pre-bulk loaded all index structures at once after a reboot (taking roughly 80 seconds) and then performed compression. In this setting, a whole human genome can be compressed within 11 seconds with 7 threads.

We think that this bulk-load scenario is actually quite realistic, as scientists often want to compress/decompress sets of genomes prior/after analysis.

### Decompression Statistics

For all tests - independent of the index block size - we obtained an average decompression time of 22 seconds. Further parallelization did not improve these values, since the decompression algorithm is I/O-bound. During the compression around 3.1 GB for the decompressed genome are written on the hard disk. Given that we measured the write speed of the hard disk with 150 MB/s, it seems hard to further optimize the decompression time. We have also tried to write only compressed FASTA files on the disk. Then, however, the decompression takes several minutes, because the (self-referential) compression is CPU-bound. The decompression times for competitors are similar, i.e. RLZopt achieves 130 MB/s and GDC up to 150 MB/s. We are convinced that for most decompressor implementations the actual limitation is the hard disk write speed.

## Conclusions

We have proposed an adaptive referential compression schemes for genomes. The compression speed can be controlled by varying the amount of main memory for the compressor. Our variant with lowest main memory footprint (12 MB) achieves similar compression rates and compression speeds, while using almost two orders of magnitude less main memory. Our greedy variant using 9 GB of main memory is 2-3 times as fast as the best known variant. Compression speed can be further improved by massive parallelization on different machines.

Further work should be done on improving the compression ratio. For instance, it would be possible to find an efficient encoding for inverse complement matches or approximate matches.

Investigations on inter-species referential compression is challenging. So far, referential compression only works well, if input and reference belong to the same species. The development of a multi-species reference sequence would allow for multi purpose genome compression algorithms. Our initial experiments with human genome compression with respect to a mouse genome indicate that the matches are usually very short and the advantages of referential compression are mitigated.

## Endnotes

^1^http://www2.informatik.hu-berlin.de/∼wandelt/blockcompression^2^ftp://ftp.1000genomes.ebi.ac.uk/vol1/ftp/release/20110521/^3^ftp://ftp.sanger.ac.uk/pub/dmc/yeast/latest

## Competing interests

The authors declare that they have no competing interests.

## Authors’ contributions

All authors have contributed equally to the main text. Sebastian Wandelt has implemented the presented algorithms on C++. All authors read and approved the final manuscript.

## References

[B1] Consortium IHGS Initial sequencing and analysis of the human genome Nature 2001 409 6822 860 921 10.1038/35057062 11237011

[B2] Schadt EE Turner S Kasarskis A A window into third-generation sequencing Human Mol Genet 2010 19 R2 R227 R240 [http://dx.doi.org/10.1093/hmg/ddq416] 10.1093/hmg/ddq416 20858600

[B3] International Cancer Genome Consortium Data Portal–a one-stop shop for cancer genomics data Database : the journal of biological databases and curation 2011 2011 0 bar026 [http://dx.doi.org/10.1093/database/bar026] 21930502 10.1093/database/bar026 PMC3263593

[B4] Kahn SD On the future of genomic data Science 2011 331 6018 728 729 [http://www.sciencemag.org/content/331/6018/728.abstract] 10.1126/science.1197891 21311016

[B5] Fusaro VA Patil P Gafni E Wall DP Tonellato PJ Biomedical cloud computing with amazon web services PLoS Comput Biol 2011 7 8 1 6 [https://sremote.pitt.edu:11018/login.aspx?direct=true&db=aph&AN=67016557&site=ehost-live] 10.1371/journal.pcbi.1002147 PMC3161908 21901085

[B6] Cloud computing and the DNA data race Nat Biotechnol 2010 28 7 691 693 [http://dx.doi.org/10.1038/nbt0710-691] 10.1038/nbt0710-691 20622843 PMC2904649

[B7] The case for cloud computing in genome informatics Genome Biol 2010 11 5 207+ [http://dx.doi.org/10.1186/gb-2010-11-5-207] 10.1186/gb-2010-11-5-207 20441614 PMC2898083

[B8] Trelles O Prins P Snir M Jansen RC Big data, but are we ready? Nat Rev Genet 2011 12 3 224 [http://dx.doi.org/10.1038/nrg2857-c1] 21301471 10.1038/nrg2857-c1

[B9] Pennisim E Will computers crash genomics? Science 2011 331 6018 666 668 [http://dx.doi.org/10.1126/science.331.6018.666] 10.1126/science.331.6018.666 21310981

[B10] Giancarlo R Scaturro D Utro F Textual data compression in computational biology: algorithmic techniques Comput Sci Rev January 2012 6 1 1 25 10.1016/j.cosrev.2011.11.001

[B11] Nalbantoglu ÖU Russell DJ Sayood K Data compression concepts and algorithms and their applications to bioinformatics Entropy 2010 12 34 52 [http://www.mdpi.com/1099-4300/12/1/34/] 20157640 10.3390/e12010034 PMC2821113

[B12] Antoniou D Theodoridis E Tsakalidis A Compressing biological sequences using self adjusting data structures10th IEEE International Conference on Information Technology and Applications in Biomedicine 2010 1 5

[B13] Pratas D Pinho AJ Rocha MP, JMC Rodríguez, Fdez-Riverola F, Valencia A Compressing the Human Genome Using Exclusively Markov Models PACBB, Volume 93 of Advances in Intelligent and Soft Computing 2011 Springer 213 220 [http://dblp.uni-trier.de/db/conf/pacbb/pacbb2011.html∖#PratasP11]

[B14] Deorowicz S Grabowski S Robust relative compression of genomes with random access Bioinformatics 2011 27 21 2979 2986 [http://dx.doi.org/10.1093/bioinformatics/btr505] 10.1093/bioinformatics/btr505 21896510

[B15] Kuruppu S Puglisi S Zobel J Chavez E, Lonardi S Relative Lempel-Ziv compression of genomes for large-scale storage and retrieval String Processing and Information Retrieval, Volume 6393 of Lecture Notes in Computer Science 2010 Berlin / Heidelberg: Springer 201 206

[B16] Kuruppu S Puglisi SJ Zobel J Relative Lempel-Ziv compression of genomes for large-scale storage and retrieval Proceedings of the 17th international conference on String processing and information retrieval, SPIRE’10 2010 Berlin, Heidelberg: Springer-Verlag 201 206 [http://dl.acm.org/citation.cfm?id=1928328.1928353]

[B17] Ukkonen E On-line construction of suffix trees Algorithmica 1995 14 249 260 [http://dx.doi.org/10.1007/BF01206331] [doi:10.1007/BF01206331] 10.1007/BF01206331

[B18] Ohlebusch E Fischer J Gog S CST++ String Processing and Information Retrieval -17th International Symposium, SPIRE 2010 2010 322 333

[B19] Kuruppu S Beresford-Smith B Conway T Zobel J Iterative dictionary construction for compression of large DNA data sets IEEE/ACM Trans Comput Biol Bioinformatics 2012 9 137 149 [http://dx.doi.org/10.1109/TCBB.2011.82] 10.1109/TCBB.2011.82 21576758

[B20] Duc Cao M Dix TI Allison L Mears C A simple statistical algorithm for biological sequence compression Proceedings of the 2007 Data Compression Conference 2007 IEEE Computer Society, Washington, DC, USA 43 52 [http://dl.acm.org/citation.cfm?id=1251981.1252877]

[B21] Christley S Lu Y Li C Xie X Human genomes as email attachments Bioinformatics 2009 25 2 274 275 [http://dx.doi.org/10.1093/bioinformatics/btn582] 10.1093/bioinformatics/btn582 18996942

[B22] Grabowski S Deorowicz S Engineering relative compression of genomes ArXiv 2011, [http://arxiv.org/abs/1103.2351] 10.1093/bioinformatics/btr505 21896510

[B23] Kreft S Navarro G LZ77-like compression with fast random access Proceedings of the 2010 Data Compression Conference, DCC ’10 2010 IEEE Computer Society, Washington, DC, USA 239 248 [http://dx.doi.org/10.1109/DCC.2010.29]

[B24] Grumbach S Tahi F Compression of DNA sequences Data Compression Conference 1993 340 350

[B25] Chen X Kwong S Li M A compression algorithm for DNA sequences and its applications in genome comparison Proceedings of the fourth annual international conference on Computational molecular biology, RECOMB ’00 2000 New York, NY, USA: ACM 107 107 [http://doi.acm.org/10.1145/332306.332352]

[B26] Manzini G Rastero M A simple and fast DNA compressor Software - Practice and Experience 2004 34 1397 1411 10.1002/spe.619

[B27] Behzadi B Le Fessant F Apostolico A, Crochemore M, Park K DNA compression challenge revisited: a dynamic programming approach Combinatorial Pattern Matching, Volume 3537 of Lecture Notes in Computer Science 2005 Berlin / Heidelberg: Springer 85 96

[B28] Cherniavsky N Ladner R Grammar-based compression of DNA sequences 2004 [Unpublished work]

[B29] Cao MD Dix TI Allison L Mears C A simple statistical algorithm for biological sequence compression Data Compression Conference 2007 0 43 52

[B30] Matsumoto T Sadakane K Imai H Biological sequence compression algorithms Genome Informatics 2000 11 43 52 11700586

[B31] Sakib MN Tang J Zheng WJ Huang CT Improving transmission efficiency of large sequence alignment/map (SAM) files PLoS ONE 2011 6 12 e28251 [http://dx.doi.org/10.1371] 10.1371/journal.pone.0028251 22164252 PMC3229529

[B32] Hsi-Yang Fritz M Leinonen R Cochrane G Birney E Efficient storage of high throughput sequencing data using reference-based compression Genome Res 2011 21 5 734 740 [http://genome.cshlp.org/content/early/2011/01/18/gr.114819.110.abstract] 10.1101/gr.114819.110 21245279 PMC3083090

[B33] BOOST C++ Libraries [ http://www.boost.org]

[B34] Ohlebusch E Fischer J Gog S CST++ SPIRE’10 2010 322 33323250716

[B35] Consortium GP A map of human genome variation from population-scale sequencing Nature 2010 467 7319 1061 1073 [http://dx.doi.org/10.1038/nature09534] 10.1038/nature09534 20981092 PMC3042601

[B36] Danecek P Auton A Abecasis G Albers CA Banks E DePristo MA Handsaker RE Lunter G Marth GT Sherry ST McVean G Durbin R 1000 Genomes Project Analysis Group The variant call format and VCFtools Bioinformatics (Oxford, England) 2011 27 15 2156 2158 [http://dx.doi.org/10.1093/bioinformatics/btr330] 10.1093/bioinformatics/btr330 PMC3137218 21653522

[B37] Kent WJ Sugnet CW Furey TS Roskin KM Pringle TH Zahler AM Haussler D The human genome browser at UCSC Genome Res 2002 12 6 996 1006 [http://www.ncbi.nlm.nih.gov/entrez/query.fcgi?cmd=Retrieve&db=PubMed&dopt=Citation&list_uids=12045153] 12045153 10.1101/gr.229102 PMC186604

